# Clinical Uses of Electricity*A lecture delivered at the Woman’s Medical College, Chicago, Nov. 2, 1888.

**Published:** 1889-01

**Authors:** Daniel R. Brower

**Affiliations:** Professor of Diseases of the Nervous System, Woman’s Medical College


CHICAGO MEDICAL

JOURNAL AND EXAMINER.

VOLUME LVIII. JANUARY, 1889. NUMBER 1.

### *THE CLINICAL USES OF ELECTRICITY.**

BY DANIEL R. BROWER, M.D.

PROFESSOR OF DISEASES OF THE NERVOUS SYSTEM, WOMAN'S MEDICAL COLLEGE.

I propose to spend this hour, ladies, in talking to you about the clinical uses of electricity. Through the kindness of Dr. McIntosh I am enabled to show you to-day the mechanical contrivances that are used for the purpose of treating patients by the various forms of electricity. There are three of these in use—the Faradic battery, the galvanic battery, and the static machine, giving us faradism, galvanism, and frictional electricity. I propose to show you these instruments and to tell you something about the clinical advantages which they present.

The first kind of electricity to which I invite your attention is the static, and I now show you this machine, the best one, I think, we have. (Fig. 1.) It is a modification of the German machine by one of our Chicago electricians, Mr. Atkinson, and is made by the McIntosh Battery and Optical Company. The current is made in part by the friction of metallic brushes on the metal discs on the revolving glass plate, and in part by induction from the stationary glass plate. The one I now show you is the 16 and 18 inch plate. By turning the handle the electricity is produced. This form of electricity is used in several ways: first, by the method of electrization, and for that purpose we place the patient on an insulated stool and have her hold a wire connected with either the hoseline or negative Leyden jar. The positive current is the more tonic and sedative. The patient takes the electrode in the hand, this switch which you see here (pointing to switch) is thrown on, and the machine is started, and the patient is soon charged with electricity. Now, this may be termed an electric bath, for it has all the advantages of one. When we charge her with the electricity, her hair, you see, stands on end; you will find the pulse accelerated, the current escaping from every part of the body, gently stimulates the end organ, scatters everywhere throughout the skin and thereby promotes the action of this great emunctory. This improvement in the circulation and activity of the skin is accompanied with a general improvement of the nutritive processes, and after the daily use of the agent you will find your patient, as a rule, increasing in weight and in vigor.

This form of electricity is sedative. In no disease is this effect better shown than in chorea. Take a patient with the choreic movements and place her upon the insulated stool you will usually notice the movements cease as she becomes charged with the positive current—and marked remedial effect will often follow its continued use.

* A lecture delivered at the Woman's Medical College, Chicago, Nov. 2, 1888.

When we want to influence particular parts of the nervous system we can do so with this machine, by drawing off the electric sparks; that is, we can concentrate the electricity at various places and draw it off so as to produce pain by the use of a sharp-pointed, or better the spheroid, electrode. But should we desire to draw off (neutralize) the electricity without pain, but in such a manner that the patient may experience a certain sensation (the electric aura), the pointed electrode should be used, but held at such a distance that no spark will leap across the interval between the patient and electrode; it draws off the electricity from the person just as a lightning-rod draws electricity from the clouds. You can in this way concentrate the current upon the head, spine, or any other part of the body without pain, and produce a soothing and tranquilizing effect often very marked and thus relieve headache and pains elsewhere about the body.

In drawing off the current in this way the electrode must be connected with the ground or with the Leyden jar opposite the one connected with patient on insulated stool, and we have here, as you see, a hook for keeping the wire or cord from touching patients. In this way we can relieve disagreeable sensations in various parts of the body. Then we can with this machine give electrical massage. Passing the current over the body we get muscular contractions equal to those produced by the faradic current, and inasmuch as it increases the muscular contractions, it determines blood to the muscular structures, and promotes, as massage does, general nutrition. This we can do without removing the clothing.

We can also use what is known as the induced current in static electricity, that is the Leyden jars become charged with electricity on the inside. The current is induced on the outside, and this induced current passes from one jar to the other by means of this switch; by moving the rods connecting with the jars so as to bring the balls near or far apart we can produce a current of greater or less intensity, and quite like the faradic. We connect the wires with the switch, place the electrodes over the muscles we desire to influence, and graduate the current as desirable and thus exercise the muscles as gently or as violently as we wish without removing the clothing.

This machine, like all other electrical appliances, requires a great deal of care and attention. It must be kept clean and dry. If the atmosphere is damp it does not work so well. The machine should be placed in a glass case, the air of which can be made dry by heat when necessary. The machine may thus be made to work well, but if the air is very damp you will find it sometimes difficult to charge your patient thoroughly, because damp air is a good conductor. Under such circumstances throw over them a rubber water-proof or macintosh, and you relieve the difficulty.

The galvanic current is another useful current in medicine. This form of electricity results from chemical action, and the galvanic battery consists essentially of two elements of different electrical potentiality and fluid that is a good conductor. (Fig. 2.) The battery before you is a McIntosh, one of the most convenient and simplest in construction I know of. It requires no mechanical ingenuity to keep such a battery in order. We have two elements, the carbon and zinc, and a fluid that is a combination of bichromate of potash, sulphuric acid, bisulphate of mercury and water. When the battery is at rest there is a space here where these elements are placed. If you desire to have the battery in action, you put the elements in the cells containing the fluid, and the current is at once established.

For increasing the current we have bifurcated cords, which I now show you. By means of these cords we can get the current we need without any shock. These cells vary their current strength from day to day. Each day the current strength grows less, but slowly. The current strength also depends somewhat upon the care and attention you give the battery, so that in order to prescribe electricity scientifically we must have some measure of current strength. For this purpose, electricians have devised a method by which we can measure these currents, and this is certainly one of the greatest advances that has been made in the therapeutic use of electricity. I can give my patient in Chicago a particular strength of current; I can tell him what the strength of that current is, and if he should move from here to New York, the physician there can give him the same current there. The instrument for measuring the current strength is the milliampère-meter. We have it on the table. We will adjust it so that our needle will be at zero, and charge the patient before you with electricity. The strength of current now passing is 40 milliampères, so that if we want our patient to have that strength of current passing through a particular portion of her body, we can designate it by the milliampère-meter.

One of the useful methods of applying galvanization is called general galvanization. This brings within the circuit all the important nerve centers, cerebral, spinal, and sympathetic. We will place the positive electrode over the forehead of the patient, while the negative electrode we will place at the nape of the neck. The current is now passing, being a little less than one milliampère. We allow the current to pass in this way for about five minutes. The current should never be interrupted while it is passing through the head. The administration of electricity to the head must be conducted at all times with the utmost care; the current should be mild so that the patient can just feel it. She is now getting one milliampère and a half through the head. When we take the electricity off the head, the current should be reduced so as not to produce any shock to the patient. If the rheostat is in the circuit with the patient, then you can graduate the current in the minutest sort of way.

The next step in the process of general galvanization is to put the positive electrode at the nape of the neck, and the negative in front and under the inner border of the sterno-cleido mastoideus muscle. There is now three-quarters of a milliampère passing through the neck. This is a very mild current, and one she appreciates.

We next pass the current through the sympathetic nervous system and through the spinal cord. To do this, we put the negative electrode over the epigastrium, which brings within the current the sympathetic nervous system as well as the spinal cord, and the positive electrode over the spinal cord, and we will increase the current until it is appreciated by the patient. Eight milliampères of current are now passing from the epigastrium to the spinal cord. This séance of general galvanization should occupy about 15 minutes. Its effects upon the nervous system are alterative, tonic, and sedative, and this kind of electricity administered with care is not at all disagreeable to patients. If the current be interrupted, the patient will have experienced shock, which should be avoided.

We may compare the galvanic electricity to a great river like the Mississippi, that flows along so smoothly and so silently that it seems to possess but little power until we attempt to arrest it; then its might is strongly manifested. But faradic electricity, like the little mountain stream, makes a good deal of noise, and seems to have a great deal of force, yet the damage it can do is comparatively slight.

Another important use for galvanic electricity is in cases of paralysis in order to produce muscular movements, and to do that we must interrupt the current. In cases of paralysis in which we desire to produce muscular movements, we may use the faradic current, or the induced current of the frictional machine, or the interrupted galvanic current. If the muscles have undergone a certain amount of degeneration they will not contract under the influence of the first or second but may under the third; and when we are treating paralyzed muscles we must use, if we desire to benefit the patient, a current that will produce these contractions. If the faradic current does not produce muscular contractions, then we use the interrupted galvanic current, and the current we use under these circumstances is the least current that will produce the contractions. If we have a paralyzed arm, for example, and the faradic current or the induced current of the static machine will not produce the necessary contractions, we place the negative electrode of the galvanic current over motor points of the muscle, and interrupt the current; then we have muscular contractions.

Every now and then patients will be brought to you with infantile paralysis, and you say you must use electricity. The mother will say, "We have been using electricity for months without any benefit." You investigate and find she is using the faradic battery. You test the muscles, and there is not the slightest response to the faradic current; consequently months of valuable time have been thrown away by using an agent powerless for any good. This is a common occurrence in my experience. You take such cases and apply the interrupted galvanic current, and you will find it may require a powerful current to get the feeblest sort of muscular contraction. If you give such a patient the electricity daily you will notice that gradually a less and less current is necessary, and that probably after a while the faradic current will produce muscular contractions, and then you can continue the treatment with the faradic current. What is true of infantile paralysis is also true of other paralyses; you must always use a current that will produce contraction, and if no current will produce the result then the case is hopeless.

Galvanic electricity has another important use in medicine; it may be used for illuminating purposes in the examination of the cavities of the body, and by this electric illuminator we can do this much more satisfactorily than by any other means. This is an electric light illuminator devised by Dr. McIntosh for clinical purposes. Suppose we want to look at this patient's throat, we can illuminate the cavity of the mouth. If we want to look into the nose, vagina, rectum, etc., we can illuminate these cavities successfully by this electric illuminator.

I next invite your attention to the faradic battery. The faradic current is dependent primarily upon chemical action going on the same as in the other battery. This inducing current under modification passes through a coil of 150 feet of wire that interrupts it about 10,000 times per minute. The modification thus produced in the current gives it much increased effect upon sensory end organs and undegenerated muscular tissue. The faradic battery gives two currents, primary and secondary. The primary is produced as above; the secondary is induced in a coil of wire in close proximity to the first coil, and differs from the primary current in having no polarity, that is with each 10,000 interruption the polarity of the electrode changes, now positive, now negative. It also differs in its more irritating effect upon the sensory and motor nerves, producing mechanical effect when the primary fails.

The galvanic current passes through the patient's body with only a sensation of warmth, unless too strong a current be used. This faradic current, on the contrary, produces a marked irritating effect upon the sensory and motor nervous system. Where the sensory system is in a condition of anæsthesia, for example, the effects of this current are much more advantageous than the other. By passing a dry electrode, such as this faradic brush, over the skin, you stimulate the peripheral ends of the sensory and motor nerves, and in that way may stimulate the centers in the brain and spinal cord by reflex action, and benefit the whole organization.

In locomotor ataxia, in anæsthesia, and in neurasthenia the faradic brush is often of therapeutic value. The faradic current is also used in cases of paralysis in which the muscles will respond. Here its value is as great as the galvanic, and the convenience of its administration is much greater.

In using the galvano-faradic currents we often have occasion to use a rheostat. It is a very important addition in the use of electricity. It enables you to graduate the current with a surprising degree of fineness and exactness. You have some patients who are exceedingly sensitive to the electric current, consequently it must be carefully graduated. This rheostat consists of a glass jar containing water, with three metallic plates, one of which moves up and down between the other two, and in that way increasing and diminishing the resistance to the current. We will gradually increase resistance, making the current so exceedingly slight that it can scarcely be felt by the patient, and then by very gradually, almost imperceptibly, decreasing it, we can often use a current of greater strength than we could otherwise use, and on the most sensitive patient.

Here is a box of electrodes so shaped as to suit various parts of the body, such as the urethra, uterus, vagina, rectum, ear, tongue, etc. Here is an abdominal one, adapted for the treatment of congestions and chronic inflammations of the uterus and its annexa. The treatment of uterine tumors by electricity is becoming a matter of very considerable importance. The electrode which you see is made of animal membrane, very pliable, and is intended to fit upon the surface of the abdomen.

To succeed in the practice of medicine in the neurological department you must study carefully the use of these various forms of electricity and the care of these various applications.

